# Association between the Plasma-Glycosylated Hemoglobin A1c/High-Density Lipoprotein Cholesterol Ratio and Carotid Atherosclerosis: A Retrospective Study

**DOI:** 10.1155/2021/9238566

**Published:** 2021-11-10

**Authors:** Xiangming Hu, Wei Li, Chenyang Wang, Haotian Zhang, Haoyu Lu, Guang Li, Yingling Zhou, Haojian Dong

**Affiliations:** ^1^The Second School of Clinical Medicine, Southern Medical University, Guangzhou, 510515 Guangdong, China; ^2^Department of Cardiology, Guangdong Cardiovascular Institute, Guangdong Provincial Key Laboratory of Coronary Heart Disease Prevention, Guangdong Provincial People's Hospital, Guangdong Academy of Medical Sciences, Guangzhou, 510080 Guangdong, China; ^3^Department of Cardiology, Guangdong Provincial People's Hospital, Zhuhai Hospital (Zhuhai Golden Bay Center Hospital), Zhuhai, 519040 Guangdong, China

## Abstract

**Background:**

Diabetes mellitus (DM) and dyslipidemia are the main risk factors for atherosclerosis. Elevated glycosylated hemoglobin A1c (HbA1c) and reduced high-density lipoprotein cholesterol (HDL-C) are associated with the progression of atherosclerosis. The aim of this study is at exploring the relationship between the HbA1c/HDL-C ratio and atherosclerosis evaluated using carotid artery intima-media thickness (cIMT) and carotid artery plaque.

**Methods:**

In this retrospective study, we enrolled 1304 patients who had multiple cardiovascular risk factors or symptoms of suspected coronary artery disease. cIMT and carotid artery plaque were measured using ultrasonography. Logistic regression was used to explore the correlation between the HbA1c/HDL-C ratio and cIMT or carotid artery plaque. We used restricted cubic spline curves to assess nonlinear relationships between the HbA1c/HDL-C ratio and cIMT or carotid artery plaque.

**Results:**

With increased quartiles of HbA1c/HDL-C, patients had higher cIMT and a greater carotid plaque burden. After adjusting for other relevant clinical covariates, patients with the highest HbA1c/HDL-C ratio (quartile 4 (Q4)) had a 2.88 times (95% confidence interval (CI): 2.02–4.10, *P* < 0.001) more abnormal mean cIMT, 3.72 times (95% CI: 2.55–5.44, *P* < 0.001) more abnormal maximum cIMT, and 2.58 times (95% CI: 1.70–3.91, *P* < 0.001) greater carotid artery plaque burden compared with patients who had the lowest HbA1c/HDL-C ratio (Q1). Moreover, the association of HbA1c/HDL-C with atherosclerosis remained significant in a subsample of patients with and without DM.

**Conclusion:**

As a novel compound indicator for evaluating blood glucose homeostasis and dyslipidemia, the HbA1c/HDL-C ratio was positively correlated with carotid atherosclerosis evaluated using the mean and maximum cIMT as well as the carotid artery plaque burden.

## 1. Introduction

Atherosclerosis, a multifactorial and systemic disease process [1, 2], can lead to cardiovascular diseases (CVD) such as coronary artery disease, ischemic stroke, and peripheral artery disease. Carotid artery intima-media thickness (cIMT) and carotid artery plaque are simple, noninvasive, and clinically accessible ultrasound markers that have been widely regarded as a surrogate for carotid atherosclerosis and a strong predictor of future CVD [3–6]. Therefore, the influence of risk factors on the development of carotid atherosclerosis can be measured by cIMT and carotid artery plaque.

The risk factors contributing to atherosclerosis are diverse, such as diabetes mellitus (DM) and dyslipidemia [7]. The relationship between DM and atherosclerosis has been widely established in clinical research and molecular research [8, 9]. cIMT gradually increases during the deterioration of glucose homeostasis from normal glucose tolerance to DM [10]. Glycosylated hemoglobin A1c (HbA1c) plays an important role in monitoring glucose control, even in patients without DM [11, 12]. Many clinical studies have described the positive relationship between HbA1c and cIMT, emphasizing the recognition of atherosclerosis development at an early stage using blood glucose monitoring [13–15]. As for dyslipidemia, in the past few decades, clinical studies and meta-analyses have consistently reported that high-density lipoprotein cholesterol (HDL-c) levels are negatively correlated with the risk of atherosclerotic CVD [16, 17]. The role of HDL-C in lipid metabolism (promoting reverse cholesterol transport), inflammation (tempering inflammatory mediator), and antithrombosis determines the close connection between low HDL-C and progression of atherosclerosis [18, 19]. However, the metabolic processes of blood glucose and lipids interact with each other. A post hoc analysis in the Stop Atherosclerosis in Native Diabetics Study (SANDS) suggested a negative correlation between HbA1c and the achievement of blood lipid goals [20]. The presence of DM eliminates the vascular protective effect of HDL-C, and poor blood glucose control is an independent risk factor for low HDL-C in DM [21, 22]. In turn, HDL-C can reduce blood glucose in patients with DM by increasing plasma insulin [23–25].

As a single index of DM or lipids for the evaluation of atherosclerosis status may be insufficient, comprehensively considering the effects of both blood glucose control and HDL-C can avoid the inadequacy of a single index. The HbA1c/HDL-C ratio is proposed as an easily available index related to atherosclerosis. To our knowledge, however, there is no research focusing on this comprehensive ratio and atherosclerosis. We hypothesized that the HbA1c/HDL-C ratio is related to subclinical atherosclerosis measured using the cIMT and carotid artery plaque via ultrasonography.

## 2. Materials and Methods

### 2.1. Study Population

**Figure 1:**
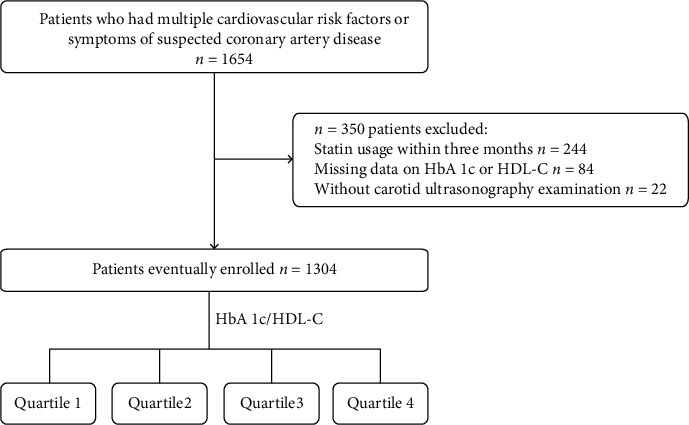
Flow chart.

This retrospective study included 1654 consecutive patients with multiple cardiovascular risk factors or symptoms of suspected coronary artery disease in Guangdong Provincial People's Hospital from September 2014 to September 2015. We collected information on demographic features, health behaviors, medical history (stroke, hypertension, diabetes, and chronic kidney disease), laboratory tests, and carotid ultrasound examinations from the electronic medical records. We excluded 244 patients who had used statins within the past 3 months, 84 patients who had missing data of HbA1c or HDL-C, and 22 patients without information of carotid ultrasonography examination (Figure 1).

This study was conducted in accordance with the Declaration of Helsinki. The study was approved by the Ethics Committee of Guangdong Provincial People's Hospital, and all patients provided their verbal informed consent.

### 2.2. Measurement

Carotid ultrasonography examination was performed manually by experienced sonographers, who were unaware of participants' baseline characteristics and laboratory results, using a GE Vivid E95 (GE Healthcare, Milwaukee, WI, USA) and 7.5–12 MHz phased array probe. Two sonographers measured cIMT and carotid artery plaque and determined the final results together. If there was any discrepancy, a third sonographer would be consulted and the results would be determined through discussion. Carotid atherosclerosis was defined as abnormal cIMT and/or presence of carotid artery plaque [4–6].

The region of interest for cIMT measurement was located at the far wall of the bilateral carotid arteries proximal to the bifurcation, with ≥10 mm of plaque-free lesions on each side. cIMT was defined as the distance between the interface of the lumen-intima and media-adventitia. The mean cIMT was taken as the average of the cIMT values of the left and right carotid arteries. Maximum cIMT was taken as the larger cIMT value between the left and right carotid arteries. We defined abnormal cIMT as mean cIMT or maximum cIMT values ≥ 1 mm [26, 27]. We also assessed the carotid artery plaque at three different locations: the common carotid artery, carotid bifurcation, and internal carotid artery on both the left and right sides. Carotid artery plaque was defined as a focal structure that encroaches into the arterial lumen at least 0.5 mm or 50% of the surrounding cIMT value or demonstrates a thickness > 1.5 mm as measured from the media-adventitia interface to the intima-lumen interface [28]. The carotid plaque score was calculated as the sum of the thickest plaque values on both the left and right sides.

### 2.3. Laboratory Examination

HbA1c was detected using the D-10™ Hemoglobin A1c Program (Bio-Rad) via high-pressure liquid chromatography. Blood cell counts were conducted using a Sysmex-XE5000 via the impedance technology. HDL-C, LDL-C, total cholesterol (TC), triglyceride (TG), apolipoprotein A (ApoA), apolipoprotein B (ApoB), fasting blood glucose, and creatinine were measured using a Beckman AU5800 spectrophotometer via colorimetry or immunoturbidimetry.

### 2.4. Covariates

Hypertension was diagnosed according to the European Society of Cardiology guidelines as systolic blood pressure ≥ 140 mmHg and/or diastolic blood pressure ≥ 90 mmHg, which is equivalent to a 24 h ambulatory blood pressure monitoring average of ≥130/80 mmHg or a home blood pressure monitoring average of ≥135/85 mmHg for two measurements over at least 3 days [29]. DM was diagnosed based on the presence of diabetes. If the patient had a negative history of diabetes, fasting blood glucose ≥ 7.0 mmol/L (126 mg/dL) and hemoglobin A1c ≥ 6.5% or positive oral glucose tolerance test (2 h plasma glucose ≥ 11.1 mmol/L (200 mg/dL)) or random plasma glucose ≥ 11.1 mmol/L (200 mg/dL) were adopted to define DM according to the European Society of Cardiology guidelines [30]. Smoking was defined as previous smoking, and alcohol consumption was defined as a previous drinking habit.

### 2.5. Statistical Analysis

Descriptive statistics were conducted using Kruskal–Wallis *H* tests and chi-square or Fisher's exact tests for continuous and dichotomous variables, respectively. We explored the relationship between the HbA1c/HDL-C ratio and abnormal cIMT or carotid artery plaque using logistic regression. The regression results are reported according to HbA1c/HDL-C ratio quartiles (as a categorical variable) using the lowest quartile (Q1) as reference. Odds ratios (ORs) with 95% confidence intervals (CIs) were calculated for the associations. We adjusted personal characteristics and healthy behaviors (age, sex, smoking, alcohol consumption, and history of hypertension) in different models to further evaluate the relationship. LDL-C, white blood cell counts, and creatinine levels were also adjusted in another model, because lipid levels, chronic low-grade inflammatory responses, and renal function affect glycometabolism and atherosclerosis. We used restricted cubic spline curves to visually assess the relationships between the HbA1c/HDL-C ratio (as a continuous variable) and abnormal cIMT or carotid artery plaque. ORs and 95% CIs were derived from restricted cubic spline regression, with knots placed at the 25th, 50th, 75th, and 95th percentiles of the distribution of the HbA1c/HDL-C ratio. The reference value of the HbA1c/HDL-C ratio was the median of the reference group.

The proportion of missing data in the sample for analysis was not greater than 5%; missing data were interpolated using the mean value. Associations with *P* < 0.05 (two sided) were considered to be statistically significant. All of the analyses were performed using Stata 15.0 (StataCorp LLC, College Station, TX, USA), R version 3.6.1 (The R Project for Statistical Computing, Vienna, Austria), and EmpowerStats (X&Y Solutions Inc., Boston, MA, USA).

## 3. Results

### 3.1. Patient Population

A total of 1304 patients with complete data were included in this retrospective study (including 897 men and 407 women). The mean age (standard deviation) was 63 (11) years. The median HbA1c/HDL-C ratio was 5.85 (interquartile range (IQR) 4.78–7.30). Baseline information of the study population is shown in Table 1. According to quartiles of the HbA1c/HDL-C ratio, patients were divided into four groups (Q1–Q4). Patients with the highest HbA1c/HDL-C ratio (Q4) mostly comprised men and had higher values of HbA1c, fasting blood glucose, cIMT, and prevalence of carotid artery plaque, as well as lower HDL-C and ApoA. The HbA1c/HDL-C ratio was positively correlated with hypertension and white blood cells. Baseline information of the study population grouped by the presence or absence of DM is shown in Supplemental Table 1 and Supplemental Table 2.

### 3.2. HbA1c/HDL-C Ratio and cIMT or Carotid Artery Plaque

**Figure 2:**
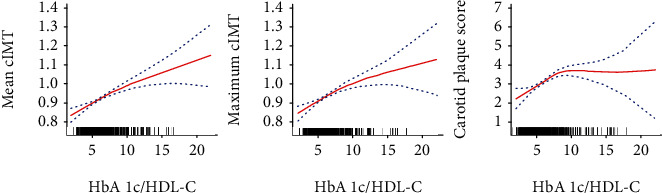
Dose–response relationship between the HbA1c/HDL-C ratio and carotid atherosclerosis. The area between the two dotted lines represents the 95% confidence interval. (a) Mean cIMT (*P* for linear trend: <0.001); (b) maximum cIMT (*P* for linear trend: <0.001); (c) carotid plaque score (*P* for linear trend: <0.001). HbA1c: hemoglobin Alc; HDL-C: high-density lipoprotein cholesterol; cIMT: carotid intima-media thickness.

**Figure 3:**
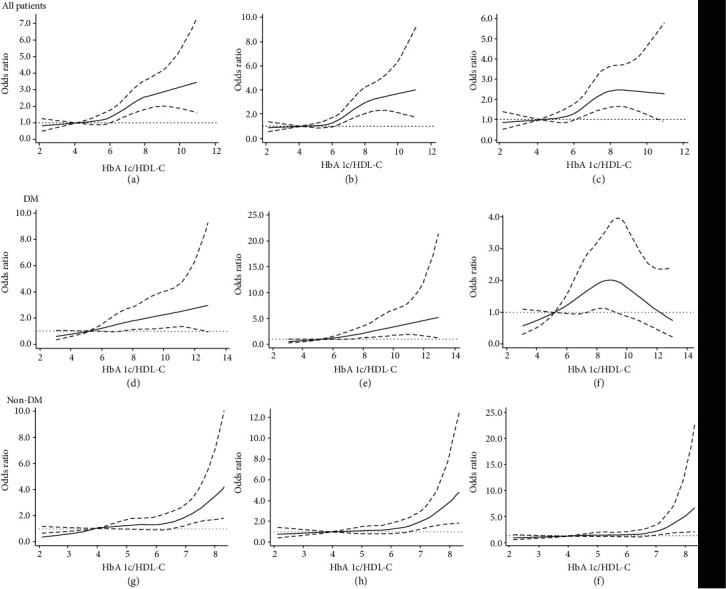
Association of the HbA1c/HDL-C ratio with carotid atherosclerosis. (a) Abnormal mean cIMT in all patients; (b) abnormal maximum cIMT in all patients; (c) carotid artery plaque in all patients; (d) abnormal mean cIMT in patients with DM; (e) abnormal maximum cIMT in patients with DM; (f) carotid artery plaque in patients with DM; (g) abnormal mean cIMT in patients without DM; (h) abnormal maximum cIMT in patients without DM; (i) carotid artery plaque in patients without DM. Odds ratios and 95% confidence intervals (CIs) were adjusted for the same variables as in model 4 (Table 2). The reference value of the HbA1c/HDL-C ratio is the median of the reference group. The area between the two dotted lines represents the 95% CI. Owing to the small sample and large 95% CI, the highest 4% of patients are not shown in the figure. HbA1c: hemoglobin Alc; HDL-C: high-density lipoprotein cholesterol; cIMT: carotid intima-media thickness; DM: diabetes mellitus.

As shown in Figure 2, the mean cIMT and maximum cIMT showed an upward trend with an increased HbA1c/HDL-C ratio (*P* < 0.001).  Table 2 presents results for the relationship between the HbA1c/HDL-C ratio and abnormal cIMT or carotid artery plaque. In the unadjusted model, the OR (95% CI) for quartile 4 (Q4) of the HbA1c/HDL-C ratio was 2.55 (1.86–3.51) for abnormal mean cIMT, 3.22 (2.29–4.52) for abnormal maximum cIMT, and 2.47 (1.69–3.60) for carotid artery plaque compared with the lowest quartile. In the age- and sex-adjusted models, Q4 showed 2.47 times (1.78–3.45) more abnormal mean cIMT, 3.20 times (2.25–4.57) more abnormal maximum cIMT, and 2.50 times (1.68–3.71) greater carotid artery plaque burden than patients with an HbA1c/HDL-C ratio in Q1. After further adjusting for smoking, alcohol consumption, and hypertension history, the ORs (95% CI) of Q4 were 2.32 (1.66–3.25) for the risk of abnormal mean cIMT, 2.95 (2.05–4.21) for the risk of abnormal maximum cIMT, and 2.30 (1.54–3.45) for carotid artery plaque, as compared with those of Q1. In the fully adjusted model, Q4 showed 2.88 times (2.02–4.10) abnormal mean cIMT, 3.72 times (2.55–5.44) abnormal maximum cIMT, and 2.58 times (1.70–3.91) greater carotid artery plaque burden in comparison with those in patients who had an HbA1c/HDL-C ratio in Q1. In the fully adjusted model, for every 1-unit increase in the HbA1c/HDL-C ratio, the risk was increased by 20% (OR:1.20, 95% CI: 1.13–1.28) for abnormal mean cIMT, by 24% (OR: 1.24, 95% CI: 1.16–1.33) for abnormal maximum cIMT, and by 13% (OR: 1.13, 95% CI: 1.05–1.21) for carotid artery plaque. The restrictive cubic spline curve showed that, after full adjustment for the same variables as in model 4 (Table 2), the HbA1c/HDL-C ratio had an S-shaped relationship with abnormal average cIMT (Figure 3(a)), abnormal maximum cIMT (Figure 3(b)), and carotid artery plaque (Figure 3(c)).

In patients with DM, after multivariable adjustment for the risk factors included in model 4, the HbA1c/HDL-C ratio was significantly correlated with abnormal mean cIMT (OR: 2.87, 95% CI: 1.55–5.32) and abnormal maximum cIMT (OR: 4.00, 95% CI: 2.00–8.02) but the relationship with carotid artery plaque did not reach a significant level (OR: 1.36, 95% CI: 0.68–2.71) (Table 3). Restricted cubic splines showed a linear relationship between the HbA1c/HDL-C ratio and abnormal cIMT (Figures 3(d) and 3(e)), but the HbA1c/HDL-C ratio and carotid artery plaque had an inverted U-shaped relationship (Figure 3(f)). In patients without DM, after multivariable adjustment, the HbA1c/HDL-C ratio was significantly correlated with abnormal mean cIMT (OR: 2.02, 95% CI: 1.30–3.15), abnormal maximum cIMT (OR: 1.92, 95% CI: 1.23–2.99), and carotid artery plaque (OR: 2.31, 95% CI: 1.39–3.86) (Table 4). The restricted cubic spline curve showed a J-shaped relationship between the HbA1c/HDL-C ratio and abnormal cIMT or carotid artery plaque (Figures 3(g)–3(i)).

### 3.3. Subgroup Analysis

Subgroup analysis showed that the above covariates (age, sex, smoking, alcohol consumption, and hypertension history) did not change the relationship between the HbA1c/HDL-C ratio and abnormal cIMT or carotid artery plaque (*P* for interaction > 0.05; Supplemental Table 3).

## 4. Discussion

In the present study, we proposed a composite indicator of blood glucose homeostasis and dyslipidemia, the HbA1c/HDL-C ratio, and demonstrated the relationship between this ratio and subclinical carotid atherosclerosis. We observed a positive correlation between HbA1c/HDL-C levels and abnormal mean/maximum cIMT as well as carotid artery plaque burden in patients with a high risk of CVD, independent of traditional clinical risk factors. More importantly, the relationship between HbA1c/HDL-C and abnormal cIMT remained in a subsample of patients with and without DM. HbA1c/HDL-C may be an important biomarker for clinical evaluation of atherosclerosis progression. This article was previously reported in the Preprint platform (2021, Research Square) [31].

Atherosclerosis is a slow, gradually progressing disease that begins in childhood, but there are potential risk factors that can accelerate its development such as poor glycemic control and low HDL-C [17, 32]. DM patients showed increased arterial stiffness and degenerated artery compliance, which worsened carotid atherosclerosis [33]. Amelioration of postprandial hyperglycemia in patients with type 2 DM could prevent the progression of carotid atherosclerosis, which indicates the importance of blood glucose homeostasis [34]. The extent of glycemic control, sensitively reflected by HbA1c, is related to CVD complications in patients with DM [35–37]. Observational studies have shown that HbA1c levels are significantly related to cIMT and carotid plaques, and long-term stable HbA1c levels are of great significance for preventing subclinical coronary atherosclerosis in patients with type 2 DM [13, 38]. Moreover, using HbA1c as an evaluation indicator, a randomized controlled trial compared intensive blood glucose control with conventional treatment, thereby proving that intensive blood glucose control can reduce the progression of CVD in DM patients [39]. Low HDL-C is an important manifestation of dyslipidemia. Clinical studies have consistently shown that low HDL-C is an independent risk factor for atherosclerotic CVD [17, 40]. A meta-analysis involving 21,000 people concluded that low HDL-C levels is associated with elevated cIMT, independent of LDL-C levels. Even in patients who are treated aggressively with statins to decrease LDL-C, HDL-C is still a major predictor of CVD development [41]. In addition, a study synthesizing three cohorts has verified that ApoA levels cannot provide additional predictive information over HDL-C for CVD, highlighting the important and independent role of HDL-C [42]. Therefore, HbA1c and HDL-C are useful indicators that reflect DM and dyslipidemia, respectively, which are closely related to the progression of atherosclerosis. Our study puts forth a new marker, the HbA1c/HDL-C ratio, which was found to be significantly associated with abnormal cIMT and carotid artery plaque after adjustment for other relevant clinical covariates. The present findings were consistent with those of previous studies on HbA1c and HDL-C [38–41]. Furthermore, the HbA1c/HDL-C ratio takes into account the relative state of blood glucose control and lipid levels. In recent years, studies have found that blood glucose homeostasis has an impact on blood lipid metabolism and vice versa. The presence of DM eliminated the protective effect of HDL-C on the progression of atherosclerosis, and furthermore, HbA1c was found to be negatively correlated with the efficacy of lipid-lowering therapy [20, 22]. In contrast, HDL-C has potent antidiabetic properties via increasing insulin sensitivity and *β*-cell function, reflecting its role in regulating blood glucose homeostasis [23–25]. In this study, the HbA1c/HDL-C ratio marker could comprehensively reflect blood glucose homeostasis and dyslipidemia, which can be used to monitor progression of atherosclerosis.

The mechanisms of deterioration of blood glucose homeostasis and dyslipidemia leading to atherosclerosis are multifaceted. Hyperglycemia increases inflammatory response and mitochondrial oxidative stress, directly resulting in vascular endothelial dysfunction [9]. A reduction in HDL-C activates local inflammation and endothelial thrombosis, increases endothelial cell apoptosis, and slows down vascular repair [43]. Inflammation caused by hyperglycemia and low HDL-C mediates the immune system disorder, disrupting the immune balance [44]. With the double attack of increasing HbA1c and decreasing HDL-C, the progression of atherosclerosis will rapidly accelerate. From this point of view, the HbA1c/HDL-C ratio could reflect the relative status of blood glucose and serum lipids as well as the extent of damage to the arterial endothelium and inflammation, which in turn can explain the rationality of the HbA1c/HDL-C ratio as a marker of atherosclerosis progression through the above pathophysiological mechanism. This shows that comprehensive management of comorbidities of glycometabolic disorder and dyslipidemia is an effective way to prevent atherosclerosis [45].

Another finding of our study was that in a subsample with and without DM, the HbA1c/HDL-C ratio was still associated with abnormal cIMT. Blood glucose homeostasis as reflected by HbA1c is not only applicable in the population with DM but also useful in those without diabetes. A recent cohort study showed that the use of HbA1c can better identify subclinical atherosclerosis in individuals without DM, on the top of traditional CVD prediction models [12]. Bobbert et al. found that in patients without DM, HbA1c had a stronger correlation with cIMT than fasting or 2 h glucose levels [46]. Because blood glucose homeostasis in people without DM will also affect subclinical atherosclerosis, HbA1c levels also require attention. Therefore, the application of the proposed new marker, the HbA1c/HDL-C ratio, for individuals without DM may have an important role in primary prevention among this population.

The present research has several limitations. First, this was a single-center study; our findings should be extrapolated carefully to other populations with different demographic characteristics. Second, the cross-sectional study design prevented us from prospectively identifying a causal relationship between the long-term HbA1c/HDL-C ratio and development of atherosclerosis. Third, in patients with DM, the dose–response relationship between the HbA1c/HDL-C ratio and carotid plaque score showed that with increased HbA1c/HDL-C ratio, total carotid artery plaque thickness was increased. Although there was no significant correlation between the HbA1c/HDL-C ratio and the presence of carotid artery plaque when the HbA1c/HDL-C ratio was at high level, these still tended to be positively correlated. The reason may be an insufficient sample size in both the DM subsample and quartile groups.

## 5. Conclusions

As a new indicator for evaluating blood glucose homeostasis and dyslipidemia, the elevated HbA1c/HDL-C ratio was found to be associated with the mean and the maximum cIMT, as well as the occurrence of carotid artery plaque, independent of conventional risk factors. These associations remained in patients with and without DM. Our study findings suggest that the HbA1c/HDL-C ratio may be useful for monitoring high-risk patients in the primary prevention of atherosclerosis.

## Figures and Tables

**Table 1:** Baseline information according to quartiles of the HbA1c/HDL-C ratio.

Characteristics	Normal control	Carotid atherosclerosis	P-value
Number of patients	*n* = 220	*n* = 1084	
Age (years)	58 ± 11	64 ± 10	<0.001
Male sex	130 (59.1%)	767 (70.8%)	<0.001
Hypertension	95 (43.2%)	672 (62.0%)	<0.001
DM	49 (22.3%)	401 (37.0%)	<0.001
Smoking	61 (27.7%)	375 (34.6%)	0.049
Alcohol consumption	11 (5.0%)	70 (6.5%)	0.414
HbA1c (%)	5.8 (5.5-6.2)	6.1 (5.7-6.8)	<0.001
Fasting blood glucose	5.6 ± 1.5	6.0 ± 2.0	0.016
TG (mmol/L)	1.5 ± 0.8	1.6 ± 1.3	0.223
TC (mmol/L)	4.5 ± 1.1	4.5 ± 1.3	0.698
ApoA (mmol/L)^†^	1.3 ± 0.3	1.2 ± 0.3	<0.001
ApoB (mmol/L)^†^	0.8 ± 0.2	0.8 ± 0.2	0.048
LDL-C (mmol/L)	2.5 ± 1.0	2.7 ± 1.1	0.094
HDL-C (mmol/L)	1.2 ± 0.3	1.1 ± 0.3	<0.001
Creatinine (mmol/L)	80.7 ± 32.2	93.0 ± 60.4	0.003
WBC (×10^9^/L)	7.2 ± 2.1	7.5 ± 2.2	0.027

Data shown as mean ± standard deviation or median (Q1–Q3) or *n* (%). ^†^Missing data were interpolated using the mean value. DM: diabetes mellitus; HbA1c: hemoglobin Alc; TG: triglyceride; TC: total cholesterol; ApoA: apolipoprotein A; ApoB: apolipoprotein B; LDL-C: low-density lipoprotein cholesterol; HDL-C: high-density lipoprotein cholesterol; WBC: white blood cells.

**Table 2:** OR (95% CIs) of abnormal cIMT and carotid artery plaque by quartiles of the HbA1c/HDL-C ratio.

	HbA1c/HDL-C					
	Each 1-unit increase	Q1 (<4.78)	Q2 (4.78–5.85)	Q3 (5.85–7.30)	Q4 (>7.30)	*P* for trend
Abnormal mean cIMT (≥1 mm)						
Model 1^†^	1.17 (1.11–1.23)	Ref.	1.10 (0.81–1.50)	1.41 (1.04–1.92)	2.55 (1.86–3.51)	<0.001
Model 2^†^	1.17 (1.10–1.23)	Ref.	1.13 (0.82–1.55)	1.45 (1.05–2.00)	2.47 (1.78–3.45)	<0.001
Model 3^†^	1.16 (1.09–1.22)	Ref.	1.10 (0.79–1.50)	1.38 (1.00–1.91)	2.32 (1.66–3.25)	<0.001
Model 4^†^	1.20 (1.13–1.28)	Ref.	1.12 (0.81–1.56)	1.54 (1.10–2.16)	2.88 (2.02–4.10)	<0.001
Abnormal maximum cIMT (≥1 mm)						
Model 1^†^	1.20 (1.13–1.28)	Ref.	1.16 (0.85–1.58)	1.48 (1.08–2.02)	3.22 (2.29–4.52)	<0.001
Model 2^†^	1.21 (1.14–1.28)	Ref.	1.20 (0.88–1.66)	1.55 (1.12–2.15)	3.20 (2.25–4.57)	<0.001
Model 3^†^	1.19 (1.12–1.26)	Ref.	1.15 (0.83–1.59)	1.45 (1.04–2.02)	2.95 (2.05–4.21)	<0.001
Model 4^†^	1.24 (1.16–1.33)	Ref.	1.19 (0.85–1.65)	1.63 (1.16–2.30)	3.72 (2.55–5.44)	<0.001
Carotid artery plaque						
Model 1^†^	1.12 (1.05–1.19)	Ref.	1.25 (0.89–1.75)	1.54 (1.09–2.18)	2.47 (1.69–3.60)	<0.001
Model 2^†^	1.12 (1.05–1.20)	Ref.	1.34 (0.94–1.91)	1.69 (1.17–2.45)	2.50 (1.68–3.71)	<0.001
Model 3^†^	1.11 (1.03–1.18)	Ref.	1.27 (0.89–1.82)	1.58 (1.09–2.30)	2.30 (1.54–3.45)	<0.001
Model 4^†^	1.13 (1.05–1.21)	Ref.	1.29 (0.90–1.86)	1.68 (1.14–2.45)	2.58 (1.70–3.91)	<0.001

Data were shown as OR (95% CI) which was evaluated using logistic regression models. ^†^Model 1: unadjusted; model 2: adjusted for age and sex; model 3: further adjusted for smoking, alcohol consumption, and history of hypertension; model 4: further adjusted for LDL-C, white blood cell count, and creatinine level; HbA1c: hemoglobin Alc; HDL-C: high-density lipoprotein cholesterol; cIMT: carotid intima-media thickness; Ref: reference; LDL-C: low-density lipoprotein cholesterol; CI: confidence interval.

**Table 3:** OR (95% CIs) of abnormal cIMT and carotid artery plaque by quartiles of the HbA1c/HDL-C ratio in patients with DM (*n* = 450).

	HbA1c/HDL-C					
	Each 1-unit increase	Q1 (<5.98)	Q2 (5.98–7.38)	Q3 (7.38–9.07)	Q4 (>9.07)	*P* for trend
Abnormal mean cIMT (≥1 mm)						
Model 1^†^	1.13 (1.04–1.22)	Ref.	1.63 (0.96–2.79)	1.70 (1.00–2.90)	2.23 (1.29–3.86)	0.005
Model 2^†^	1.13 (1.04–1.23)	Ref.	1.56 (0.90–2.70)	1.68 (0.97–2.90)	2.29 (1.28–4.08)	0.006
Model 3^†^	1.16 (1.09–1.22)	Ref.	1.53 (0.88–2.67)	1.68 (0.97–2.92)	2.23 (1.25–4.01)	0.008
Model 4^†^	1.13 (1.04–1.23)	Ref.	1.65 (0.94–2.91)	1.85 (1.05–3.26)	2.87 (1.55–5.32)	0.001
Abnormal maximum cIMT (≥1 mm)						
Model 1^†^	1.17 (1.06–1.28)	Ref.	1.79 (1.02–3.15)	2.17 (1.21–3.86)	2.80 (1.53–5.15)	0.001
Model 2^†^	1.17 (1.07–1.29)	Ref.	1.72 (0.97–3.08)	2.17 (1.20–3.92)	2.94 (1.55–5.57)	0.001
Model 3^†^	1.17 (1.06–1.29)	Ref.	1.68 (0.93–3.02)	2.16 (1.19–3.94)	2.83 (1.48–5.41)	0.001
Model 4^†^	1.22 (1.10–1.35)	Ref.	1.82 (0.99–3.34)	2.44 (1.30–4.56)	4.00 (2.00–8.02)	<0.001
Carotid artery plaque						
Model 1^†^	1.03 (0.94–1.12)	Ref.	1.38 (0.73–2.59)	2.02 (1.02–3.99)	1.22 (0.66–2.28)	0.342
Model 2^†^	1.04 (0.95–1.14)	Ref.	1.38 (0.72–2.65)	2.11 (1.05–4.22)	1.37 (0.71–2.64)	0.208
Model 3^†^	1.04 (0.94–1.14)	Ref.	1.35 (0.68–2.60)	2.09 (1.04–4.22)	1.30 (0.67–2.54)	0.259
Model 4^†^	1.04 (0.95–1.15)	Ref.	1.36 (0.70–2.63)	2.10 (1.04–4.27)	1.36 (0.68–2.71)	0.222

Data were shown as OR (95% CI) which was evaluated using logistic regression models. ^†^Model 1: unadjusted; model 2: adjusted for age and sex; model 3: further adjusted for smoking, alcohol consumption, and history of hypertension; model 4: further adjusted for LDL-C, white blood cell count, and creatinine level; HbA1c: hemoglobin Alc; HDL-C: high-density lipoprotein cholesterol; cIMT: carotid intima-media thickness; Ref: reference, DM: diabetes mellitus; LDL-C: low-density lipoprotein cholesterol; CI: confidence interval.

**Table 4:** OR (95% CIs) of abnormal cIMT and carotid artery plaque by quartiles of the HbA1c/HDL-C ratio in patients without DM (*n* = 854).

	HbA1c/HDL-C					
	Each 1-unit increase	Q1 (<4.41)	Q2 (4.41–5.23)	Q3 (5.23–6.38)	Q4 (>6.38)	*P* for trend
Abnormal mean cIMT (≥1 mm)						
Model 1^†^	1.11 (1.02–1.22)	Ref.	1.27 (0.87–1.86)	1.16 (0.79–1.69)	1.67 (1.14–2.44)	0.020
Model 2^†^	1.10 (1.00–1.22)	Ref.	1.24 (0.83–1.85)	1.25 (0.84–1.87)	1.61 (1.07–2.44)	0.032
Model 3^†^	1.09 (0.99–1.20)	Ref.	1.24 (0.83–1.85)	1.22 (0.81–1.82)	1.56 (1.03–2.36)	0.052
Model 4^†^	1.16 (1.05–1.29)	Ref.	2.02 (0.81–1.56)	1.54 (1.10–2.16)	2.02 (1.30–3.15)	0.002
Abnormal maximum cIMT (≥1 mm)						
Model 1^†^	1.13 (1.03–1.24)	Ref.	1.11 (0.76–1.62)	1.11 (0.76–1.62)	1.63 (1.10–2.40)	0.021
Model 2^†^	1.13 (1.02–1.25)	Ref.	1.09 (0.73–1.62)	1.21 (0.81–1.81)	1.60 (1.06–2.44)	0.025
Model 3^†^	1.11 (1.00–1.23)	Ref.	1.08 (0.72–1.61)	1.15 (0.77–1.73)	1.52 (1.00–2.32)	0.051
Model 4^†^	1.18 (1.06–1.31)	Ref.	1.09 (0.73–1.65)	1.25 (0.83–1.90)	1.92 (1.23–2.99)	0.004
Carotid artery plaque						
Model 1^†^	1.20 (1.07–1.34)	Ref.	1.42 (0.93–2.16)	1.05 (0.70–1.58)	2.01 (1.29–3.14)	0.015
Model 2^†^	1.22 (1.08–1.38)	Ref.	1.46 (0.94–2.28)	1.20 (0.78–1.86)	2.10 (1.29–3.40)	0.011
Model 3^†^	1.20 (1.06–1.36)	Ref.	1.41 (0.89–2.21)	1.12 (0.72–1.74)	1.98 (1.21–3.25)	0.027
Model 4^†^	1.25 (1.10–1.43)	Ref.	1.43 (0.90–2.27)	1.17 (0.74–1.83)	2.31 (1.39–3.86)	0.007

## Data Availability

The data analyzed in this study can be obtained from the corresponding author with a reasonable request.

## Abbreviations

DM: Diabetes mellitus
HbA1c: Glycosylated hemoglobin A1c
HDL-C: High-density lipoprotein cholesterol
LDL-C: Low-density lipoprotein cholesterol
cIMT: Carotid artery intima-media thickness
CVD: Cardiovascular diseases
TC: Total cholesterol
TG: Triglyceride
ApoA: Apolipoprotein-A
ApoB: Apolipoprotein-B
OR: Odds ratio
CI: Confidence interval
IQR: Interquartile range
Q: Quantile.

## Supplementary Materials

Supplementary Materials Supplemental Table 1: baseline information according to quartiles of the HbA1c/HDL-C ratio in patients with DM (*N* = 450). Supplemental Table 2: baseline information according to quartiles of the HbA1c/HDL-C ratio in patients without DM (*N* = 854). Supplemental Table 3: subgroup analysis of abnormal cIMT or carotid artery plaque in prespecified and exploratory subgroups.
