# Supplementary material for: Association between the Plasma-Glycosylated Hemoglobin A1c/High-Density Lipoprotein Cholesterol Ratio and Carotid Atherosclerosis: A Retrospective Study
Source: J Diabetes Res. 2021 Nov 10;2021:9238566. doi: 10.1155/2021/9238566 (PMC8598339; doi:10.1155/2021/9238566)
Supplement: Supplementary Materials — Supplemental Table 1: baseline information according to quartiles of the HbA1c/HDL-C ratio in patients with DM (N = 450). Supplemental Table 2: baseline information according to quartiles of the HbA1c/HDL-C ratio in patients without DM (N = 854). Supplemental Table 3: subgroup analysis of abnormal cIMT or carotid artery plaque in prespecified and exploratory subgroups. [file 9238566.f1.pdf]

Association between plasma glycosylated hemoglobin A1c/high-density lipoprotein cholesterol ratio and carotid atherosclerosis: a retrospective study

Supplemental Table 1. Baseline information according to quartiles of HbA1c/HDL-C ratio in patients with DM (N=450)

Characteristics	HbA1c/HDL-C ratio				
	Q1 (<5.18)	Q2 (5.18–6.77)	Q3 (6.77–8.23)	Q4 (>10.73)	P value
Number of patients	112	113	113	112	
Age, years	66.4 (9.6)	65.0 (9.4)	64.6 (10.2)	62.0 (11.4)	0.013
HbA1c, %	6.6 (6.3–7.0)	6.8 (6.5–7.3)	7.4 (6.9–8.2)	8.9 (7.6–9.9)	<0.001
TG, mmol/L	1.5 (0.8)	1.9 (2.0)	1.8 (1.3)	2.1 (1.8)	0.015
TC, mmol/L	4.9 (1.1)	4.5 (1.1)	4.3 (1.4)	3.9 (1.3)	<0.001
ApoA, mmol/L†	1.5 (0.3)	1.1 (0.2)	1.1 (0.2)	1.0 (0.2)	<0.001
ApoB, mmol/L†	0.8 (0.2)	0.8 (0.2)	0.8 (0.3)	0.7 (0.2)	0.032
LDL-C, mmol/L	2.8 (1.0)	2.6 (0.9)	2.6 (1.1)	2.2 (0.9)	<0.001
HDL-C, mmol/L	1.3 (0.2)	1.0 (0.1)	0.9 (0.1)	0.8 (0.2)	<0.001
Creatinine, mmol/L	85.9 (46.8)	97.2 (59.9)	91.8 (64.2)	114.9 (121.9)	0.037
Mean-cIMT, mm	1.0 (0.8–1.0)	1.0 (0.8–1.1)	1.0 (0.9–1.1)	1.0 (0.9–1.1)	0.001
Maximum cIMT, mm	1.0 (0.8–1.1)	1.0 (0.9–1.2)	1.1 (1.0–1.2)	1.1 (1.0–1.2)	0.008
Male sex	54 (48.2%)	81 (71.7%)	76 (67.3%)	88 (78.6%)	<0.001
Smoking	20 (17.9%)	42 (37.2%)	32 (28.3%)	43 (38.4%)	0.002
Alcohol consumption	6 (5.4%)	8 (7.1%)	4 (3.5%)	4 (3.6%)	0.556
Hypertension	76 (67.9%)	80 (70.8%)	74 (65.5%)	77 (68.8%)	0.860
Carotid plaque score	3.4 (0.8–4.7)	3.9 (1.6–5.0)	3.9 (2.2–5.0)	3.7 (1.5–5.1)	0.239
Carotid artery plaque	84 (75.0%)	91 (80.5%)	97 (85.8%)	88 (78.6%)	0.229
WBC, ×109/L	7.3 (2.0)	7.9 (2.4)	7.8 (2.1)	7.9 (2.3)	0.156
Fasting blood glucose	5.8 (5.2–6.7)	6.1 (5.3–7.3)	6.9 (5.8–8.6)	8.0 (6.3–10.2)	<0.001

Data were shown as mean ± standard deviation or median (Q1–Q3) or n (%).

†Missing data were interpolated using the mean value.

HbA1c: hemoglobin Alc, HDL-C: high-density lipoprotein cholesterol, TG: triglyceride, TC: total cholesterol, ApoA: apolipoprotein A, ApoB: apolipoprotein B, LDL-C: low-density lipoprotein cholesterol, cIMT: carotid intima-media thickness, WBC: white blood cells

Supplemental Table 2. Baseline information according to quartiles of HbA1c/HDL-C ratio in patients without DM (N=854)

Characteristics	HbA1c/HDL-C ratio				P value
	Q1 (<4.41)	Q2 (4.41–5.23)	Q3 (5.23–6.38)	Q4 (>6.38)	
Number of patients	213	214	214	213	
Age, years	64.1 (10.2)	62.9 (10.7)	60.4 (10.8)	61.9 (11.1)	0.003
HbA1c, %	5.6 (5.4–5.9)	5.7 (5.4–6.0)	5.8 (5.6–6.1)	5.9 (5.7–6.1)	<0.001
TG, mmol/L	1.2 (0.8)	1.4 (0.7)	1.6 (0.9)	1.8 (1.4)	<0.001
TC, mmol/L	5.0 (1.3)	4.7 (1.1)	4.5 (1.3)	4.0 (1.1)	<0.001
ApoA, mmol/L†	1.6 (0.3)	1.3 (0.2)	1.1 (0.2)	0.9 (0.2)	<0.001
ApoB, mmol/L†	0.8 (0.3)	0.8 (0.2)	0.8 (0.3)	0.8 (0.2)	0.102
LDL-C, mmol/L	2.8 (1.1)	2.8 (1.0)	2.7 (1.2)	2.4 (0.9)	<0.001
HDL-C, mmol/L	1.5 (0.2)	1.2 (0.1)	1.0 (0.1)	0.8 (0.1)	<0.001
Creatinine, mmol/L	77.5 (22.6)	83.3 (25.2)	87.1 (35.3)	102.0 (61.1)	<0.001
Mean-cIMT, mm	0.9 (0.8–1.0)	0.9 (0.8–1.0)	0.9 (0.8–1.0)	1.0 (0.8–1.1)	0.026
Maximum cIMT, mm	1.0 (0.8–1.1)	1.0 (0.8–1.1)	1.0 (0.8–1.1)	1.0 (0.9–1.1)	0.025
Male sex	107 (50.2%)	148 (69.2%)	156 (72.9%)	187 (87.8%)	<0.001
Smoking	58 (27.2%)	66 (30.8%)	81 (37.9%)	94 (44.1%)	0.001
Alcohol consumption	9 (4.2%)	19 (8.9%)	14 (6.5%)	17 (8.0%)	0.251
Hypertension	101 (47.4%)	116 (54.2%)	119 (55.6%)	124 (58.2%)	0.141
Carotid plaque score	2.3 (0.0–4.4)	3.2 (0.2–4.5)	3.0 (0.0–4.3)	3.6 (1.6–4.8)	0.004
Carotid artery plaque	144 (67.6%)	160 (74.8%)	147 (68.7%)	172 (80.8%)	0.007
WBC, $\times 10^9$/L	7.0 (2.2)	7.1 (1.9)	7.5 (2.3)	7.7 (2.3)	0.004
Fasting blood glucose	4.9 (4.6–5.3)	5.0 (4.6–5.5)	5.0 (4.7–5.6)	5.0 (4.7–5.6)	0.021

Data were shown as mean ± standard deviation or median (Q1–Q3) or n (%).
†Missing data were interpolated using the mean value.
HbA1c: hemoglobin Alc, HDL-C: high-density lipoprotein cholesterol, TG: triglyceride, TC: total cholesterol, ApoA: apolipoprotein A, ApoB: apolipoprotein B, LDL-C: low-density lipoprotein cholesterol, cIMT: carotid intima-media thickness, WBC: white blood cells

Supplemental Table 3. Subgroup analysis of abnormal cIMT or carotid artery plaque in prespecified and exploratory subgroups

Abnormal mean cIMT					
Subgroups	HbA1c/HDL-C ratio				P for interaction
	Q1 (<4.78)	Q2 (4.78–5.85)	Q3 (5.85–7.30)	Q4 (>7.30)	
Age ⩾ 65 years					0.860
Yes	Ref.	1.1 (0.7–1.8)	1.7 (1.1–2.7)	2.6 (1.6–4.2)	
No	Ref.	1.2 (0.8–1.8)	1.4 (0.9–2.1)	2.7 (1.7–4.2)	

Sex					0.983
Male	Ref.	1.1 (0.8–1.7)	1.4 (0.9–2.0)	2.4 (1.6–3.6)	
Female	Ref.	0.9 (0.6–1.6)	1.3 (0.8–2.3)	1.3 (0.8–2.3)	
Smoking					0.210
Yes	Ref.	1.1 (0.6–1.9)	1.2 (0.7–2.0)	2.0 (1.1–3.4)	
No	Ref.	1.1 (0.8–1.6)	1.5 (1.0–2.2)	2.8 (1.9–4.2)	
Alcohol consumption					0.941
Yes	Ref.	1.9 (0.5–6.4)	1.2 (0.4–4.2)	3.9 (0.9–17.6)	
No	Ref.	1.1 (0.8–1.5)	1.4 (1.0–2.0)	2.5 (1.8–3.5)	
Hypertension					0.683
Yes	Ref.	0.9 (0.6–1.4)	1.3 (0.9–2.0)	2.1 (1.4–3.3)	
No	Ref.	1.3 (0.8–2.1)	1.3 (0.8–2.2)	2.7 (1.7–4.5)	
Abnormal maximum cIMT					
Age ⩾ 65 years					0.654
Yes	Ref.	1.1 (0.7–1.8)	1.9 (1.1–3.1)	2.9 (1.7–4.9)	
No	Ref.	1.3 (0.8–2.0)	1.4 (0.9–2.2)	3.7 (2.3–5.9)	
Sex					0.713
Male	Ref.	1.1 (0.7–1.6)	1.4 (0.9–2.0)	2.9 (1.9–4.4)	
Female	Ref.	1.2 (0.7–2.0)	1.4 (0.8–2.5)	3.5 (1.8–6.6)	
Smoking					0.713
Yes	Ref.	1.1 (0.6–2.0)	1.3 (0.8–2.4)	2.9 (1.6–5.3)	
No	Ref.	1.2 (0.8–1.7)	1.5 (1.0–2.2)	3.3 (2.2–5.0)	
Alcohol consumption					0.981
Yes	Ref.	1.8 (0.5–6.0)	1.9 (0.5–6.4)	3.1 (0.7–13.8)	
No	Ref.	1.1 (0.8–1.6)	1.5 (1.1–2.0)	3.2 (2.3–4.6)	
Hypertension					0.766
Yes	Ref.	1.1 (0.7–1.7)	1.6 (1.0–2.5)	2.9 (1.9–4.7)	
No	Ref.	1.1 (0.7–1.8)	1.2 (0.7–1.8)	3.1 (1.8–5.2)	
Carotid artery plaque					
Age ⩾ 65 years					0.206
Yes	Ref.	1.1 (0.6–2.0)	1.8 (0.9–3.4)	1.7 (0.9–3.1)	
No	Ref.	1.5 (1.0–2.3)	1.7 (1.1–2.6)	3.4 (2.1–5.5)	
Sex					0.510
Male	Ref.	1.1 (0.7–1.7)	1.4 (0.9–2.2)	2.1 (1.3–3.4)	
Female	Ref.	1.4 (0.8–2.4)	1.6 (0.9–2.9)	2.9 (1.4–6.0)	
Smoking					0.476
Yes	Ref.	1.1 (0.6–2.1)	1.5 (0.8–3.0)	1.8 (0.9–3.6)	
No	Ref.	1.3 (0.9–1.9)	1.5 (1.0–2.2)	2.7 (1.7–4.4)	
Alcohol consumption					0.336
Yes	Ref.	0.1 (0.0–1.3)	0.2 (0.0–2.2)	0.8 (0.0–13.4)	
No	Ref.	1.3 (1.0–1.9)	1.6 (1.1–2.3)	2.6 (1.8–3.8)	
Hypertension					0.921
Yes	Ref.	1.1 (0.7–1.9)	1.4 (0.8–2.3)	2.2 (1.3–3.7)	
No	Ref.	1.2 (0.8–1.9)	1.4 (0.9–2.4)	2.3 (1.3–4.0)	

Data were shown as OR (95%CI).

cIMT: carotid intima-media thickness, HbA1c: hemoglobin Alc, HDL-C: high-density lipoprotein cholesterol
